# Supplementary material for: Learning curve and surgical outcome of robotic assisted colorectal surgery with ERAS program
Source: Sci Rep. 2022 Nov 29;12:20566. doi: 10.1038/s41598-022-24665-w (PMC9709162; doi:10.1038/s41598-022-24665-w)
Supplement: Supplementary file 2 — Supplementary Information 2. [file 41598_2022_24665_MOESM2_ESM.zip › RAL Raw data-Ñ[▒K/133/HS_ñw╝╨░O▒Kñσ.pdf]

正式摘要(電子簽章：000000233036323)

W92 / W92 ☐ 英文標題(注意:無英文姓名-請補病人基本檔!) ☐【讀取】憑證

臺中榮民總醫院

出院病歷摘要

轉入號： 轉出號：

[redacted]

(8)流水編號	
(9)入院日期	110/12/14 (2021) 直腸外科 W92 - 37
(10)轉科日期	
(11)出院日期	110/12/23 (2021) 住院天數計 9 日

診斷

- 入院：1. Adenocarcinoma of sigmoid colon at 35cm FAV, c T1N0M0, s/p endoscopic mucosal resection on 2021/11/17
  2. Uterine leiomyoma, s/p myomectomy on 2010/10/1
- 出院：1. Adenocarcinoma of sigmoid colon at 35cm FAV, c T1N0M0,
  s/p endoscopic mucosal resection on 2021/11/17,
  s/p Robotic AR on 2021/12/15
  pT1N0(0/15), MD, AI-, PI-,
  2. Uterine leiomyoma, s/p myomectomy on 2010/10/1
  3. gastritis, HP+

主訴

- constipation and abdominal pain

病史

- This 48 years old female patient has a medical history of
  1. Uterine leiomyoma, s/p myomectomy on 2010/10/18
  2. polyp at 35cm FAV and A colon in CMUH on 2018
  According to her, she received health exam in CMUH on 2018. Poly at 35 cm FAV was noted that time. Recently, due to constipation and abdominal pain, she went to our CRS OPD. Colonscopy with endoscopic mucosal resection was performed. Reoprt showed adenocarcinoma. Abdominal CT on 2021/12/08 showed uterin myoma. Under the impression of Adenocarcinoma of sigmoid colon , c T1N0M0, the patient was admitted for surgery.

體檢發現

- 2018/9 A colon and S colon polyp.
  patho: 30 cm: adeno ca. 70 cm TVA.
  2021/12 CT: uterin myoma.
  - General appearance :
    - easy looking , conscious clear , without cardiopulmonary distress
    - Vital signs自動帶入date 體溫(BT) 脈搏(P) 呼吸(R) 血壓(BP) 身高(BH) 體重(BW) BMI
      20211214 35.7℃ 68次/分 18次/分 111/63mmHg 162.4cm 71.3kg 27.0
  - HEENT :
    - Eyes: no anemic conjunctiva, no icteric sclera, normal EOM
    - ENT: no otorrhea, no rhinorrhea, no erythematous mucosa, no injected tonsil, no deformity
  - Neck: supple, freely movable, trachea midline,no deformity, no engorged jugular vein , no palpable thyroid or lymph node enlargement
  - Chest:
    - Inspection: Grossly normal, symmetric and fully expansion, no accessory muscle used, no operation scar, no spider angiomata, no gynecomastia
    - Palpation: no palpable mass or nodule, no subcutaneous emphysema, no tenderness
    - Ausculation: clear breathing sound
  - Heart:
    - Percussion: normal heart size
    - Ausculation: regular heart beat, normal S1, S2, no gallop, no murmur
  - Abdomen:
    - Inspection: in
      - flat contour, symetrically, no scar, no stria, no dilated veins, no rashes
    - Ausculation: normoactive bowel sound, no abdominal bruits
    - palpation: soft, no muscle guarding or rigidity, no local tenderness, no rebounding pain, no palpable mass, no palpable pulsation
    - Percussion: no shifting dulllness, no hepatomegaly, no splenomagaly
  -

Peripheral pulsation :	CA	BA	RA	FA	PA	PTA	DPA
Right side :	++	++	++	++	++	++	++/+++
Left side :	++	++	++	++	++	++	++/+++

  - Genito-anus: no tenderness or palpable mass lesion or rectal shelf, normal external genitalia, normal anal tone, brown stool

CRS CONDITION

- CEA: 0.429 ng/ml
- Imaging studies(Sono , CT of abdomen and chest Films):
  - 檢驗項目:CT ABDOMEN-SURVEY 報告日期:1101213
    主 訴：
    Result:Y Finding: Imaging Report Form for Colorectal Carcinoma (AJCC 8th) 1. Imaging Date: 20211208 Imaging Modality, Imaging by CT scan Imaging Protocol: CT of abdomen with IV and oral contrast enhancement: 2. Tumor location / Size Location: Sigmoid Size: Non-measurable 3. Tumor invasion Tumor cannot be visualized in this imaging study 4. Regional nodal metastasis No 5. Distant metastasis (In this study) No or Equivocal 6. Other findings: > A case of colon cancer for staging > The primary lesion at S-colon cannot be identified in the study. Please correlate with colonoscopy. > Some low attenuation lesions are noted over bilateral lobes of liver, favor hepatic cysts. Please correlate with abdominal US. > The gallbladder, biliary tract, pancreas, spleen, bilateral adrenal glands, bilateral kidneys, and bilateral lower lung fields shows no obvious abnormal > No obvious enlarged lymph nodes are noted over retroperitonium, masentary, and bilateral inginal fossa > Several heterogeneous enahnced nodules within uterine cavity, favored uterine myomas. Please correlate with GYN condition. > Presence of the right ovarian cystic lesion > No obvious bony lesions are noted Impression: 1. The primary lesion at S-colon cannot be identified in the study. Please correlate with colonoscopy. 2. For other details, please see the above descriptions.
-
  - Lung

手術日期及方法

- 手術日期及方法20211215 1. Robotic (XI) assisted anterior resection
  2. Intraoperative colonoscopy : (-). Already s/p colonscopy EMR and tatto on 2021/12
  3. High ligation (+) : At IMA root. Left colic artery and IMV were also ligated
  4. Take down from the splenic flexure : (-)
  5. Robotic endoGIA (45-4.3) X2 + EEA (29mm) were used
  6. Proximal cut end: 10 cm, distal cut end: 10 cm
  7. No drainage tube was inserted
  8. Hyalobarrier gel was applied upon paraumbilical trocar wound
  9. 2-0 prolene was used to lift the uterus out from pelvis
  10. Indocyanine green agent was administered for checking the blood supply status of the remaining colon

住院治療經過

-

- After admission, pre-op survye was done andshowed no contraindication for op. Robot AR was performed on 12/15. After op, dizziness and vomittng was complained. But after PCA off, sympotms was improved. She can tolerated soft diet. Stitch and CVC was removed on 12/22. Under the stable condition, she was discharged and arranged OPD f/u.

合併症

一般檢查

- 累積報告 :

DATE	TIME	ALB	TP	BIL.T	BIL.D	ALKP	AST	ALT	LDH	AMY	LIPASE	NH3	Ferritin	UA
1101215	1607			0.39	0.11		17	12						
1101220	0922	3.5					18	11						

DATE	TIME	WBC	RBC	HGB	HCT	MCV	PLT	BLST	NEUT	BAND	SEG	LYM	MON	EOS	BAS
1101215	1608	12950		12.5	37.7		293		91.5			5.5	2.8	0.0	0.2
1101220	0921	5000	3.42	10.1	30.3	88.6	281		54.6			36.0	5.8	3.2	0.4

DATE	TIME	NA	K	CL	BUN	CREAT	eGFR	CA	Mg	P	CRP	ESR	PCT	D-Dimer	LACTATE
1101215	1607	138	3.9	107	6	0.72		8.2							
1101220	0922	141	3.6	109	6	0.61	111.26	7.8							

- 放射線報告 :檢驗項目 **:CHEST AP VIEW (PORTABLE)** 報告日期 **:1101216**

主 訴 :
Result:N
Finding:
> Some bowel gas and fecal material are noted.
> Increased interstitial infiltration over bilateral lower lung field.
> On nasogastric tube, on EKG lead, on central line
> Mild left CP angle blunting, favor pleural effusion or pleural change.
Impression:
As above description.

檢驗項目 **:CT ABDOMEN-SURVEY** 報告日期 **:1101213**
主 訴 :
Result:Y
Finding:
Imaging Report Form for Colorectal Carcinoma (AJCC 8th)
1. Imaging Date: 20211208
Imaging Modality, Imaging by
CT scan
Imaging Protocol: CT of abdomen with IV and oral contrast enhancement:
2. Tumor location / Size
Location:
Sigmoid
Size:
Non-measurable
3. Tumor invasion
Tumor cannot be visualized in this imaging study
4. Regional nodal metastasis
No
5. Distant metastasis (In this study)
No or Equivocal
6. Other findings: > A case of colon cancer for staging
> The primary lesion at S-colon cannot be identified in the study. Please correlate with colonoscopy.
> Some low attenuation lesions are noted over bilateral lobes of liver, favor hepatic cysts. Please correlate with abdominal US.
> The gallbladder, bililary tract, pancreas, spleen, bilateral adrenal glands, bilateral kidneys, and bilateral lower lung fields shows no obvious abnormal
> No obvious enlarged lymph nodes are noted over retroperitonium, masentary, and bilateral inginal fossa
> Several heterogeneous enahnced nodules within uterine cavity, favored uterine myomas. Please correlate with GYN condition.
> Presence of the right ovarian cystic lesion
> No obvious bony lesions are noted
Impression:
1. The primary lesion at S-colon cannot be identified in the study. Please correlate with colonoscopy.
2. For other details, please see the above descriptions.

特殊檢查

病理報告

- 活體組織切片病理報告 手術日期 : **110/11/17** 病理號 : **S1037605**

診斷醫師:李欣倪 報告日期:110/11/22
Pathologic diagnosis:
1. Colon, 70 cm from anal verge, "A", endoscopic biopsy —
Tubulovillous adenoma.
2. Colon, 32 cm from anal verge, "B", endoscopic biopsy —
Insufficient tissue.
3. Colon, 30 cm from anal verge, "C", endoscopic biopsy —
Adenocarcinoma, moderately differentiated, arising from tubulovillous adenoma. Tumor invades to submucosa with lymphovascular invasion.
Prognostic and predictive factor:
1. Polyp size: 0.8 cm.
2. Size of invasive carcinoma: 0.3 cm.
3. Histologic grade: Moderately differentiated.
4. Microscopic tumor extension: Submucosa.
5. Margins: (1) Deep margin: Uninvolved by invasive carcinoma, 2 mm in distance. (2) Mucosal margin: Uninvolved by invasive carcinoma.
6. Lymph-vascular invasion: Present.
7. Type of polyp which invasive carcinoma arose: Tubulovillous adenoma.
8. Additional pathological finding: Not identified.
Gross description:
The specimen consists of 1) a piece of tan soft tissue, 0.5x0.4x0.4 cm, labeled as "colon, 70 cm". 2) a piece of tan soft tissue, 0.5x0.3x0.2 cm, labeled as "colon, 32 cm". 3) a piece of tan soft tissue, 0.8x0.8x0.8 cm, labeled as "colon, 30 cm". All for section: A) specimen A B) specimen B C) specimen C.
Microscopic description:
Section A shows colon mucosa with tubulovillous glands proliferation with dysplasia. Section B shows no tissue left. Section C shows colon mucosa with invasive carcinoma in a background of tubulovillous adenoma.
Note:
1. The diagnosis is concurred in the intradepartment consensus meeting on 2021/11/19.
2. This case has been peer reviewed by two doctors.
#T-59300_2 #M-81403_2 2 1100 00CM0F
#T-59300_2 #M-82630_2 0 1100 000000

活體組織切片病理報告 手術日期 : **110/12/10** 病理號 : **S1041150**

診斷醫師:[illegible]仔璇 報告日期:110/12/15
Pathologic diagnosis:

- Uterus, endometrium, biopsy –– Disordered proliferative endometrium.
  Gross description:
  The specimen received consists of a piece of tan gray soft tissue, 1x1x0.2 cm. All for section.
  Microscopic description:
  Section shows endometrial tissue in the disordered proliferative stage.
  #T-83401_2 #M-79310_2 0 1140 000000

  活體組織切片病理報告　手術日期：**110/12/15**　病理號：**S1041896**

  診斷醫師:吳鈫瑩 報告日期:110/12/24
  Pathologic diagnosis:
  1. IMA root, dissection –– Unremarkable lymph nodes (0/3).
  2. Sigmoid colon, robot-assisted colectomy –– Status post endoscopic mucosal resection with focal xanthomatous inflammation. No residual tumor is seen. The bilateral cut ends and mesocolic lymph nodes (0/12) are free of tumor.
  3. Colon, cut end, distal, resection –– Unremarkable.
  Prognostic and predictive factor:
  (combined with previous specimen S1037605)
  1. Tumor size: 0.3 cm.
  2. Depth of invasion: Submucosa.
  3. Histologic grade: G2: Moderately differentiated.
  4. Angiolymphatic invasion: Not identified.
  5. Perineural invasion: Not identified.
  6. Tumor deposits (discontinuous extramural extension): Not identified.
  7. Macroscopic tumor perforation: Not identified.
  8. Surgical Margins:
  a) Proximal Margin: Uninvolved by invasive carcinoma.
  b) Distal Margin: Uninvolved by invasive carcinoma.
  c) Radial or Mesenteric Margin: Uninvolved by invasive carcinoma, distance of tumor from margin cannot be assessed.
  9. Lymph node status: Uninvolved (0/15).
  10. Extranodal involvement: Not applicable.
  11. Tumor regression grading S/P CCRT: No known presurgical therapy.
  12. Type of polyp in which invasive carcinoma arose: Tubulovillous adenoma.
  13. Additional pathologic findings: Not identified.
  14. Pathological TNM Stage: pT1N0 (According to the eighth edition, American Joint Committee on Cancer Staging Guidelines for Tumors).
  15. TNM descriptors: Not applicable.
  Gross description:
  The specimen consists of 1) IMA root, a piece of tan yellow soft tissue, 2.4x1.8x1.5 cm. 2) a segment of colon, 13.6 cm in length and 5.7 cm in proximal cut end circumference, 5.5 cm in distal circumference, and mesocolic soft tissue. No tumor, polypoid or ulcerative lesion is seen over the mucosa. There is a black stitch, 6.5 cm from the nearest margin. An area with black submucosal pigmentation, 2x1 cm, is seen 1 cm from the stitch. On cross section, no lesion is seen around the stitch site or pigmented area. The both cut ends are free of lesion grossly. 3) distal cut end, a piece of tan mucosa-covered soft tissue, 2.3x1.9x1 cm. Representative part for section: A) specimen 1 B) proximal cut end C) distal cut end D-E) stitch site F-G) pigmented area H) radial margin near stitch site J) specimen 3 K-L) lymph nodes of mesocolon.
  Note:
  This case has been peer reviewed by two doctors.
  #T-59470_2 #M-09413_2 0 1105 SP0000
  #T-C4560_2 #M-09183_2 0 1105 000000

其它

出院時情況

- 3.改門診治療

出院指示

藥名	劑量	劑量單位	用法	頻次	數量	數量單位	院況	天數	單位
Voren-K tab 25mg	1	TAB	PO	BIDPRN	6	TAB	TH	3	天
(□)Takepron OD tab 30mg	1	TAB	PO	QDAC	3	TAB	TH	3	天
Ultracet tab	1	TAB	PO	Q12HPRN	6	TAB	TH	3	天

出院計劃

主治醫師 2222H 林俊余 / 住院醫師 2346C 陳怡豪

正式摘要(電子簽章：**000000233036323)**

W92 / W92

doctorRank:B
